# Relative Digestibility of Breads Made with Different Leavening Agents

**Published:** 1900-02

**Authors:** Edgar Everhart

**Affiliations:** Atlanta, Ga.; Professor Chemistry Atlanta Dental College


ATLANTA

### Journal-Record of Medicine.

Successor to Atlanta Medical and Surgical Journal, Established 1855, and Southern Medical Record, Established 1870.

VOL. I. FEBRUARY, 1900. No. 11.

EDITORS { BERNARD WOLFF, M.D., DUNBAR ROY, M.D.

M. B. HUTCHINS, M.D., BUSINESS MANAGER.

PUBLISHED MONTHLY. OFFICE 305 AND 306 FITTEN BUILDING.

### ORIGINAL COMMUNICATIONS.

### RELATIVE DIGESTIBILITY OF BREADS MADE WITH DIFFERENT LEAVENING AGENTS.

BY EDGAR EVERHART, PH.D.,

Professor Chemistry Atlanta Dental College.

In the periodic wars between baking-powder companies concerning the merits of their respective products there is generally displayed almost a total lack of knowledge of the real essence of the matter, namely, of the action which the various baking-powders produce upon the digestive agents of the human body. There have been countless analyses of the powders and certificates as to their purity, as well as expressions of personal opinion as to their value; but, as far as the writer is aware, nothing has been done to elucidate the problem.

In order to throw some light upon the subject the following investigation was undertaken independent of the interests of any baking-powder company. It was thought that the best and most reliable results could be obtained by subjecting various breads to artificial digestion as nearly like the natural as possible, both quantitatively and qualitatively. No abnormal quantities either of the substance digested or of the digestive agents were used. Excessive amounts of any material introduced into the stomach are certain to bring about derangements of the various organs, and therefore care was taken to have all the conditions as near as possible natural.

Again, it did not seem essential to the investigation to make isolated experiments upon the action of the saliva, of the gastric juice and of the pancreatic juice, because in natural digestion ultimately all three must exist together, and may act in concert. The scope of the work was limited to the study of the action of saliva alone, of the action of saliva and of the gastric juice, and, finally, of the action of the saliva, of the gastric juice, and of the pancreatic juice together.

The various breads made from baking-powder were prepared under the direction of myself, while the yeast breads were bought from bakers. In selecting the samples for the work only the inside of the biscuits or bread was used, none of the crust being taken. All the samples were carefully crumbled as finely as possible, without "balling" them. In each series they were kept in air-tight flasks until all the experiments pertaining to that series were completed. The biscuits were made in every case just as they are made usually for consumption.

In the preparation of the digestive agents it was my intention to use Merck's pepsin and pancreatin, but as they were not to be obtained in this market recourse was had to Parke, Davis & Armour's preparations. The saliva used was fresh from the mouth of one person; and after a sufficient quantity was obtained, it was mixed with a certain amount of water, and an aliquot portion taken from the same for all the experiments in each series of determinations. As the analyses of gastric juice vary so much, two different solutions were made. The first contained:

| | |
|---|---|
| Water | 99.26 % |
| Pepsin and organic matter | 0.30 " |
| Free hydrochloric acid | 0.22 " |
| Alkaline chlorides | 0.20 " |
| Phosphates of calcium and magnesium | 0.02 " |

The second contained:

| | |
|---|---|
| Water | 99.44 % |
| Pepsin and organic matter | 0.30 " |
| Free hydrochloric acid | 0.02 " |
| Calcium chloride | 0.006 " |
| Sodium " | 0 14 " |
| Potassium " | 0.05 " |
| Calcium Phosphate | 0.01 " |

As no reliable analysis of the pancreatic juice is known, the following formula was used:

| | |
|---|---|
| Water | 97.60 % |
| Pancreatin and organic matter | 1.80 " |
| Phosphate of soda | 0.20 " |
| Carbonate of soda | 0.30 " |

The amounts of saliva and artificial gastric juice used on the samples subjected to the digestion were, as near as possible, proportional to the amounts secreted by the various organs of the human body that act upon the average daily amount of human food.

The actual digestion was carried on in large ovens, whose temperature was kept at 37° to 38.5° C. (98.6° to 101.3° F.) during the whole process. The breads were placed in small Erlenmeyer flasks, and the various solutions, warmed to about 38° C., were poured on to them. In all the experiments the greatest care was exercised to make all conditions exactly alike, so that strictly comparable results could be obtained.

The experiments were carried on upon five different kinds of bread, viz.: bread made with alum, cream of tartar, and phosphate baking-powders, upon soda-buttermilk biscuits, and upon yeast bread. These five kinds of bread represent the ones nearly always used.

The following method of procedure was adopted in every one of the series of experiments. Three samples of each of the five different breads were accurately weighed (3 grams being taken always), and introduced into small Erlenmeyer flasks. Fifty cc. of the warm saliva solution were poured upon the fifteen samples, which were then placed immediately in the air-bath and kept at a temperature of 37° to 38.5° C. for one hour. At the end of that time five of the flasks, each containing a different bread, were withdrawn, cooled rapidly, the volume made up to 250 cc. with cold water

(at 5° C), and an aliquot part filtered off in which the sugar was determined gravimetrically by Allihn's method.

At the time these five samples were taken from the oven, namely, at the end of the first hour, the other ten flasks received each 50 cc. of the warm artificial gastric juice, and were then returned to the air-bath for a period of three hours, at the same temperature. At the expiration of this time five of the flasks, each with a different bread, were taken out, cooled rapidly, the volume made up to 250 cc. with cold water, and an aliquot part filtered off for the sugar determination as before. The rest of the liquid was filtered off, the residue washed on the filter with cold water, then dried, and the undigested proteids determined by estimating the nitrogen according to Kjeldahl's method.

To the five remaining flasks, at the time of the withdrawal of the second set (at the end of the fourth hour), were added 50 cc. of a warm artificial pancreatic juice. These were then returned to the oven and digested for one hour longer at the same temperature. They were then withdrawn, cooled and treated as the previous set for the estimation of sugar and proteids.

During these digestions considerable differences in the physical conditions of the various breads were noted. The cream of tartar, the alum, and the soda-buttermilk breads quickly disintegrated, even under the influence of the saliva, and were converted into a very finely pulverulent state, which was a great hindrance to rapid filtration, although, no doubt, it exposed the bread more thoroughly to the action of the ferments. The phosphate bread exhibited the same condition in two of the tests, but the yeast bread retained the original form of the crumbs until after the pancreatin had acted upon it.

After the action of the pepsin, starch could not be detected in the filtered solution of any of the breads except in the case of the yeast bread. This test was made only on one of the series, and was made with iodine solution.

The following tables show the results of the various determinations seriatim. The sugar (invert sugar), as representing the first stage of the digestion of starch, was determined in the several solutions directly without heating with an acid.

### DIGESTION WITH SALIVA ALONE.

Time of digestion, 1 hour.
Three different series on three different bakings.

| | Alum. | | | Yeast. | | | Soda. | | | Cream of Tartar. | | | Phosphate. | | |
|---|---|---|---|---|---|---|---|---|---|---|---|---|---|---|---|
| | 1 | 2 | 3 | 1 | 2 | 3 | 1 | 2 | 3 | 1 | 2 | 3 | 1 | 2 | 3 |
| Sugar in solution from saliva digestion. ........ | 26 95 | 30.17 | 29.64 | 18.08 | 8.98 | 11.68 | 28.48 | 28.75 | ..... | 26.72 | 23.95 | 25 34 | 20.70 | 27.32 | 31.34 |

The figures represent percentages calculated on the breads employed, and dried at 100° C. to constant weight.

In the above table it is seen that there is not a great difference between the amounts of starch digested in any one of the four baking-powders used, except possibly in the first series of the phosphate. The discrepancy in this case may have been due to the imperfect mixing of the two separate powders which constitute the ordinary phosphate baking-powder. This imperfect mixing is quite likely to occur at any time in this particular powder, as the cook has to mix with the flour bicarbonate of soda from one package and the acid phosphate from another.

In the case of yeast bread there is a marked difference from the others. In every instance the amount of starch digested is considerably lower than with the other breads.

### DIGESTION WITH SALIVA AND GASTRIC JUICE.

Time of digestion, 1 hour with saliva alone and 3 hours with the mixed solutions.
Three different series on three different bakings.

| | Alum. | | | Yeast. | | | Soda. | | | Cream of Tartar. | | | Phosphate. | | |
|---|---|---|---|---|---|---|---|---|---|---|---|---|---|---|---|
| | 1 | 2 | 3 | 1 | 2 | 3 | 1 | 2 | 3 | 1 | 2 | 3 | 1 | 2 | 3 |
| Sugar in solution from saliva and gastric digestion. | 26.07 | 29.24 | 23.39 | 16.13 | 8.97 | 7.57 | 17.42 | 28.17 | .... | 26.37 | 21.06 | 24.00 | 19.61 | 26.78 | 27.99 |
| Proteids undigested by saliva and gastric juice, 0.02 % HCl............ | ..... | ..... | 3.02 | ..... | .... | 3.89 | ..... | ..... | 7.37 | ..... | ..... | 4.57 | ..... | ..... | 6.87 |
| Proteids undigested by saliva and gastric juice 0.22 % HCl............ | 0.75 | 0.71 | ..... | 1.51 | 1.69 | .... | 1.22 | 3.00 | .... | 0.83 | 0.80 | ..... | 1.51 | 1.51 | ..... |

At this stage of the digestion relatively the same variations of the amounts of starch converted into sugar in the different breads is seen as in the first stage with saliva alone, the yeast bread being still very much behind the others. Attention will be called later on to the decreased amount of sugar in the solution in every instance.

With regard to the proteids, the figures show the percentage undigested. The difference between the total amount of proteids in the breads and the amount given in this table represents the peptones, or the nitrogenous matter, in solution. The first two series were digested with gastric juice containing 0.22 per cent. of hydrochloric acid, the third series with that having only 0.02 per cent. It can be seen how much more energetic was the action of the pepsin in the former case. The large amount of undigested proteids in the second and third series of the soda-buttermilk bread affords an excellent illustration of the variability of that kind of bread. In its preparation, if a quantity of lactic acid (from the buttermilk) insufficient to neutralize the bicarbonate of soda is used, the reaction of the bread will be alkaline and will tend to neutralize the acid of the gastric juice and to promote indigestion. From these results it would appear that so-called soda biscuits are excellent when a sufficient quantity of buttermilk is employed, but otherwise they become very indigestible as far as the proteids are concerned. The reaction of the breads seems to have no particular influence on the digestion of the starch, but only on that of the nitrogenous substances. In the third series of the phosphate bread the high percentage of undigested proteids was probably due to an excess of soda.

DIGESTION WITH SALIVA, GASTRIC AND PANCREATIC JUICES.

Time of digestion, 1 hour with saliva alone, 3 hours with saliva and gastric juice, and 1 hour with all three digestive agents.

Three different series on three different bakings.

| | Alum. | | | Yeast. | | | Soda. | | | Cream of Tartar | | | Phosphate. | | |
|---|---|---|---|---|---|---|---|---|---|---|---|---|---|---|---|
| | 1 | 2 | 3 | 1 | 2 | 3 | 1 | 2 | 3 | 1 | 2 | 3 | 1 | 2 | 3 |
| Sugar in solution. Saliva and gastric and pancreatic digestion.... | 19.30 | 21.26 | 22.48 | 15.90 | 9.01 | 5.21 | 15.81 | 23.22 | .... | 20.20 | 19.45 | .... | 19.05 | 24.84 | 27.99 |
| Proteids undigested by saliva and gastric and pancreatic juices.... | ..... | ..... | 0.69 | ..... | .... | 0.68 | ..... | ..... | 1.22 | ..... | ..... | 0.78 | ..... | .... | 1.31 |

With this final digestion to which the breads were subjected the same variations occur as in the two previous digestions. The proteids were determined in one series only, viz.: in that one in which the small percentage of hydrochloric acid was added to the pepsin. It might be mentioned that filtration in the case of this final digestion was extremely difficult with all the breads, the residues being mucilaginous in their consistency.

Another series of determinations was made with the alum, yeast and phosphate breads to determine the amounts of unconverted starch left undigested at each of the three stages of the artificial digestion. The process was carried out in the same manner as in all the other series, except that the undissolved residue in each case, instead of being used to determine undigested proteids, was treated with hydrochloric acid to convert the starch into sugar, which was determined as usual.

DETERMINATION OF UNCONVERTED STARCH.

| | Alum. | Yeast. | Phosphate. |
|---|---|---|---|
| Starch insoluble in saliva | 18.05 | 36.99 | 18.94 |
| Starch insoluble in saliva and gastric juice | 17.29 | 25.64 | 17.28 |

The undissolved starch, after the pancreatic digestion, is not given because it was found impossible to wash the residues sufficiently to obtain reliable results.

The five following tables give all the determinations made upon each bread, including the chief component parts of that bread. The sixth table gives the averages.

BREAD MADE WITH ALUM POWDER.

| | | | | | Average |
|---|---|---|---|---|---|
| Moisture | ........ | 26.43 | 41.22 | 40.51 | 36.05 |
| Ash | ........ | 2.18 | ..... | ..... | 2.18 |
| Starch | ........ | ..... | 76.71 | ..... | 76.71 |
| Proteids | 11.31 | 11.31 | 10.88 | ..... | 11.17 |
| Sugar in saliva solution | ........ | 26.95 | 30.17 | 29.64 | 28.92 |
| Sugar in saliva and gastric juice | ........ | 26.07 | 29.24 | 23.39 | 26.23 |
| Sugar in saliva, gastric and pancreatic juices | ........ | 19.30 | 21.26 | 22.48 | 21.01 |
| Proteids undigested by saliva and gastric juice | with 0.02 % HCl | ..... | ..... | 3.02 | 3.02 |
| | with 0.22 % HCl | 0.75 | 0.71 | ..... | 0.73 |
| Proteids undigested by saliva, gastric and pancreatic juices | ........ | ..... | ..... | 0.69 | 0.69 |
| Starch undigested by saliva | 18.05 | ..... | ..... | ..... | 18.05 |
| Starch undigested by saliva and gastric juice | 17.29 | ..... | ..... | ..... | 17.29 |

BREAD MADE WITH YEAST.

| | | | | | Average |
|---|---|---|---|---|---|
| Moisture | | 40.10 | 40.13 | 37.10 | 39.11 |
| Ash | | 1.13 | ..... | ..... | 1.13 |
| Starch | | ..... | ..... | ..... | ....... |
| Proteids | 11.84 | 11.40 | 13.81 | ..... | 12.35 |
| Sugar in saliva solution | | 18.09 | 8.98 | 11.68 | 12.91 |
| Sugar in saliva and gastric juice | | 16.13 | 8.97 | 7.57 | 10.89 |
| Sugar in saliva, gastric and pancreatic juices | | 15.90 | 9.01 | 5.21 | 10.04 |
| Proteids undigested by saliva and gastric juice | with 0.02 % HCl | ..... | ..... | 3.89 | 3.89 |
| | with 0.22 % HCl | 1.51 | 1.69 | ..... | 1.60 |
| Proteids undigested by saliva, gastric and pancreatic juices | | ..... | ..... | 0.68 | 0.68 |
| Starch undigested by saliva | 36.99 | ..... | ..... | ..... | 36.99 |
| Starch undigested by saliva and gastric juice | 25.64 | ..... | ..... | ..... | 25.64 |

BREAD MADE WITH SODA-BUTTERMILK.

| | | | | | Average |
|---|---|---|---|---|---|
| Moisture | | 35.73 | 40 01 | ..... | 37.87 |
| Ash | | 1.52 | ... | ..... | 1.52 |
| Starch | | ..... | 76.71 | ..... | 76.71 |
| Proteids | 13.50 | 13.50 | 11.37 | 11.80 | 12.22 |
| Sugar in saliva solution | | 28.48 | 28.75 | ..... | 28.62 |
| Sugar in saliva and gastric juice | | 17.42 | 28 17 | ..... | 22.80 |
| Sugar in saliva, gastric and pancreatic juices | | 15 81 | 23.22 | ..... | 19.52 |
| Proteids undigested by saliva and gastric juice | with 0.02 % HCl | ..... | .... | 7.37 | 7.37 |
| | with 0.22 % HCl | 1.22 | 3.00 | ..... | 2.11 |
| Proteids undigested by saliva, gastric and pancreatic juices | | ..... | ..... | 1.22 | 1.22 |

BREAD MADE WITH CREAM OF TARTAR POWDER.

| | | | | | Average |
|---|---|---|---|---|---|
| Moisture | | 23.02 | 37.80 | ..... | 30.41 |
| Ash | | 1.72 | ..... | ..... | 1.72 |
| Starch | | ..... | ..... | ..... | 76.71 |
| Proteids | | 11.25 | 11.40 | ..... | 11.33 |
| Sugar in saliva solution | | 26.72 | 23.95 | 25.34 | 25.34 |
| Sugar in saliva and gastric juice | | 26.37 | 21.06 | 24.00 | 23.72 |
| Sugar in saliva, gastric and pancreatic juices | | 20.20 | 19.45 | ..... | 19.83 |
| Proteids undigested by saliva and gastric juice | with 0.02 % HCl | ..... | ..... | 4.57 | 4.57 |
| | with 0.22 % HCl | 0.83 | 0.80 | ..... | 0.815 |
| Proteids undigested by saliva, gastric and pancreatic juices | | ..... | ..... | 0.78 | 0.78 |

BREAD MADE WITH PHOSPHATE POWDERS.

| | | | | | Average |
|---|---|---|---|---|---|
| Moisture | | 47.99 | 50.33 | 50.12 | 49.48 |
| Ash | | 2.46 | ..... | ..... | 2.46 |
| Starch | | ..... | ..... | ..... | 76.71 |
| Proteids | | 11.38 | 10.65 | 11.40 | 11.14 |
| Sugar in saliva solution | | 20.70 | 27.32 | 31.34 | 29.79 |
| Sugar in saliva and gastic juice | | 19.61 | 26.78 | 27.99 | 24.79 |

BREAD MADE WITH PHOSPHATE POWDERS—CONTINUED.

| | | | | | Average |
|---|---|---|---|---|---|
| Sugar in saliva, gastric and pancreatic juices | ... | 19.05 | 24.84 | 27.99 | 23.96 |
| Proteids undigested by saliva and gastric juice | with 0.02 % HCl | ..... | .. . | 6.87 | 6.87 |
| | with 0.22 % HCl | 1.51 | ..... | ..... | 1.51 |
| Proteids undigested by saliva, gastric and pancreatic juices | ... | .... | ..... | 1.31 | 1.31 |
| Starch undigested by saliva | 18.94 | ..... | ..... | ..... | 18.94 |
| Starch undigested by saliva and gastric juice | 17.28 | ..... | ..... | ..... | 17.28 |

RÉSUMÉ OF AVERAGES.

| | Alum. | Yeast. | Soda. | Cream of Tartar. | Phosphate. |
|---|---|---|---|---|---|
| Moisture | 36.05 | 39.11 | 37.87 | 30.41 | 49.48 |
| Ash | 2.18 | 1.13 | 1.52 | 1.72 | 2.46 |
| Starch | 76.71 | ..... | 76.71 | 76.71 | 76.71 |
| Proteids | 11.17 | 12.35 | 12.22 | 11.33 | 11.14 |
| Sugar in saliva solution | 28.92 | 12 91 | 28.62 | 25.34 | 29.79 |
| Sugar in saliva and gastric juice | 26.23 | 10.89 | 22.80 | 23.72 | 24.79 |
| Sugar in saliva, gastric and pancreatic juices. | 21.01 | 10.04 | 19.52 | 19.83 | 23.96 |
| Proteids undigested by saliva and gastric juice—with 0.02 % HCl | 3.02 | 3.89 | 7.37 | 4.57 | 6.87 |
| with 0.22 % HCl | 0.73 | 1.60 | 1.22 | 0.815 | 1.51 |
| Proteids undigested by saliva, gastric and pancreatic juices. | 0.69 | 0.68 | 1.22 | 0.78 | 1.31 |
| Starch undigested by saliva | 18.05 | 36.99 | ..... | ..... | 18.94 |
| Starch undigested by saliva and gastric juice. | 17.29 | 25.64 | ..... | ...... | 17.28 |

In all of these tables the percentages are based on the dried breads.

An examination of the last table, the table of averages, shows that the greatest amount of sugar converted in the saliva digestion occurs in the case of the phosphate bread, with alum second, soda third, cream of tartar fourth, and last yeast. The sugar found in solution, after digesting with the other ferments, exists in approximately the same proportion except that in one instance the alum bread has more. Of unconverted starch the alum bread has least, then the phosphate, while the yeast has by far the most. With regard to the proteids, again the alum outranks the other leavening agents, with yeast bread next, then cream of tartar, and last the soda and phosphate. The low standing of the soda-buttermilk bread is no doubt due to its alkalinity, and of the phosphate to its imperfect mixing. Upon the whole, the bread made with the alum powder stands first in its digestibility, both in regard to its starch contents as well as to its proteids, while yeast bread falls far below all in its starch digestion, although its proteids seem to do better. Evidently the mineral salts, sulphate, lactate and phosphate of soda and Rochelle salt have a powerfully stimulating action upon the ferments of digestion, especially upon the ptyalin. The sulphate of soda, in particular, promotes rapid and complete digestion.

From an examination of these tables, it will be noticed that the ptyalin continues to act on the starch in connection with the gastric juice. This is seen in the case of the determinations of the undigested starch after the action of saliva and after the action of the saliva in conjunction with the pepsin.

Furthermore, it will be observed that there is a gradual disappearance of the sugar in solution as it passes through the different stages of digestion. Also, it can be seen that the sum of the starch in the residues plus the sugar in solution by no means accounts for all the starch present in the bread. If sugar (invert sugar) were the ultimate change produced by digestion, its amount should increase and be much greater after the pancreatic digestion than after that of the ptyalin. The contrary is the case in every instance.

A study of this phenomenon has been begun and will be the subject of another investigation. The work done so far indicates that a much more profound change takes place in the digestion of the carbohydrates than is usually supposed.

